# Post mortem computed tomography and core needle biopsy in comparison to autopsy in eleven bernese mountain dogs with histiocytic sarcoma

**DOI:** 10.1186/s12917-015-0544-0

**Published:** 2015-09-02

**Authors:** Franziska C. Hostettler, Dominique J. Wiener, Monika M. Welle, Horst Posthaus, Urs Geissbühler

**Affiliations:** Clinical Radiology, Department of Clinical Veterinary Medicine, Vetsuisse Faculty, University of Bern, post office box 8466, CH-3001 Bern, Switzerland; Institute of Animal Pathology, Vetsuisse Faculty, University of Bern, Länggassstrasse 122, CH-3012 Bern, Switzerland

**Keywords:** Post mortem computed tomography, Core needle biopsy, Bernese mountain dog, Histiocytic sarcoma, Autopsy

## Abstract

**Background:**

Bernese mountain dogs are reported to have a shorter life expectancy than other breeds. A major reason for this has been assigned to a high tumour prevalence, especially of histiocytic sarcoma. The efforts made by the breeding clubs to improve the longevity with the help of genetic tests and breeding value estimations are impeded by insufficiently reliable diagnoses regarding the cause of death. The current standard for post mortem examination in animals is performance of an autopsy. In human forensic medicine, imaging modalities, such as computed tomography and magnetic resonance imaging, are used with increasing frequency as a complement to autopsy. The present study investigates, whether post mortem computed tomography in combination with core needle biopsy is able to provide a definitive diagnosis of histiocytic sarcoma. For this purpose we have analysed the results of post mortem computed tomography and core needle biopsy in eleven Bernese mountain dogs. In the subsequent autopsy, every dog had a definitive diagnosis of histiocytic sarcoma, based on immunohistochemistry.

**Results:**

Computed tomography revealed space-occupying lesions in all dogs. Lesion detection by post mortem computed tomography was similar to lesion detection in autopsy for lung tissue (9 cases in computed tomography / 8 cases in autopsy), thoracic lymph nodes (9/8), spleen (6/7), kidney (2/2) and bone (3/3). Hepatic nodules, however, were difficult to detect with our scanning protocol (2/7). Histology of the core needle biopsies provided definitive diagnoses of histiocytic sarcoma in ten dogs, including confirmation by immunohistochemistry in six dogs. The biopsy samples of the remaining dog did not contain any identifiable neoplastic cells. Autolysis was the main reason for uncertain histological diagnoses.

**Conclusions:**

Post mortem computed tomography is a fast and effective method for the detection of lesions suspicious for histiocytic sarcoma in pulmonary, thoracic lymphatic, splenic, osseous and renal tissue. Optimization of the procedure regarding the scanning protocol and tissue sample size and number will improve the accuracy of the method.

## Background

Imaging diagnostic methods have been established as valuable complements to autopsy in human forensic medicine [1,2]. Especially advantageous are the non-destructive way of examination and the observer-independent archiving of objective data [3]. Computed tomography (CT) is a preferred method because of its fast way of data acquisition [4] and because it can be used conveniently to guide minimally-invasive tissue sampling [5].

In our Institute of Animal Pathology, the current standard for investigation of the cause of death in animals is an autopsy following standard protocols, considering medical history, clinical symptoms and specific differential diagnoses. Radiography is commonly used to reveal the existence and localisation of bullets and fractures within a carcass, especially in wild and companion animals found dead.

Recently, the use of advanced imaging diagnostic methods in veterinary medicine has been recommended [6, 7] and considered [6, 8–10].

Bernese mountain dogs (BMD) are reported to have a shorter life expectancy than other breeds of comparable size [11, 12]. In the Swiss population of BMD, the main reason for an early death has been assigned to a high prevalence of neoplasia, especially of lymphoma and histiocytic sarcoma (HS) [13]. A low rate of tissue sampling and a low reliability of the reported diagnoses regarding the cause of death were found in the survey study of Klopfenstein [13]. In order to find ways to improve the health status in the breed, the Swiss Club for the Bernese mountain dog intends to establish a health management program called “from the cradle to the grave”. In a representative random sample of the population, health data shall be collected throughout the life of the dogs. The cause of death will be an important information that can be derived from intra vitam, as well as post mortem (PM) examinations. Autopsies of pet animals are performed with decreasing frequency in the Institute of Animal Pathology in Bern, due to decreasing owner compliance [14, 15].

In order to find ways to improve data collection regarding the causes of death, the Swiss Club for the BMD together with Clinical Radiology and the Institute of Animal Pathology of the Vetsuisse Faculty of Bern offered owners of purebred BMD a post mortem examination, consisting of a whole body CT combined with core needle biopsy and a subsequent routine autopsy. The intention was, to test the feasibility of this minimally invasive technique and to compare the findings with the results of a routine autopsy. The examination protocols had to respect economic limits, however.

The Swiss Club for the BMD also provided international researchers with blood and tissue samples from dogs suffering from confirmed HS, to support the development of a genetic test for HS. Additionally, health data was integrated into breeding value estimations.

Over a period of one year, 33 deceased or euthanized BMD could be examined by PM-CT. Owners of 28 dogs allowed subsequent autopsy. In eleven dogs, autopsy diagnosis was HS, one of the most frequent causes of death of BMD [11–13, 16–19]. The aim of the present study was, to compare findings and diagnoses of PM-CT combined with core needle biopsy to autopsy. It was not designed to derivate breeding recommendations.

## Methods

Owners and breeders of BMD were informed via the Swiss Club for the Bernese mountain dog (breeder congress, club website) and the crematory (employees, website) about our interest in spontaneously deceased or euthanized dogs. The owners were asked to inform us as soon as possible about planned or performed euthanasia, to enable rapid transportation of the cadaver to the university. The procedure was explained to all owners before starting the PM-CT. The Veterinary Office of the Canton of Bern confirmed, that according to the Swiss Federal Welfare Act of December 16^th^ 2005, no animal experimentation licence was required. For this study, the results of eleven out of 28 cases were evaluated. The eleven dogs met the following inclusion criteria: detection of space - occupying lesions in PM-CT, performance of CT-guided biopsy and a definite diagnosis of HS in autopsy. In the eleven dogs selected for this study, the PM-CT was performed one hour to twelve days post mortem. Eight dogs were examined two to twelve hours post mortem and two dogs within 24 hours. Dog 10 was examined twelve days after death. The body of this dog had been frozen right after euthanasia and stored there until the examination.

A whole body CT was conducted by FCH with a 16 slice helical CT scanner (Brilliance 16 Slice, Philips). The examination was divided into three separate scans, the first included head, neck and front limbs, the second one the thorax, and the third one included abdomen and hind limbs. For all body regions the same settings were used, namely 120 kVp, 200 mAs and a slice thickness of 3 mm. In a few cases, specific regions were rescanned with a thinner slice thickness (e.g. bones). Interpretation of the scans was performed on a double monitor workstation (Extended Brilliance Workspace) by FCH and UG. After performance of the scans, a temperature probe was inserted into the liver to measure the inner body temperature.

**Fig. 1:**
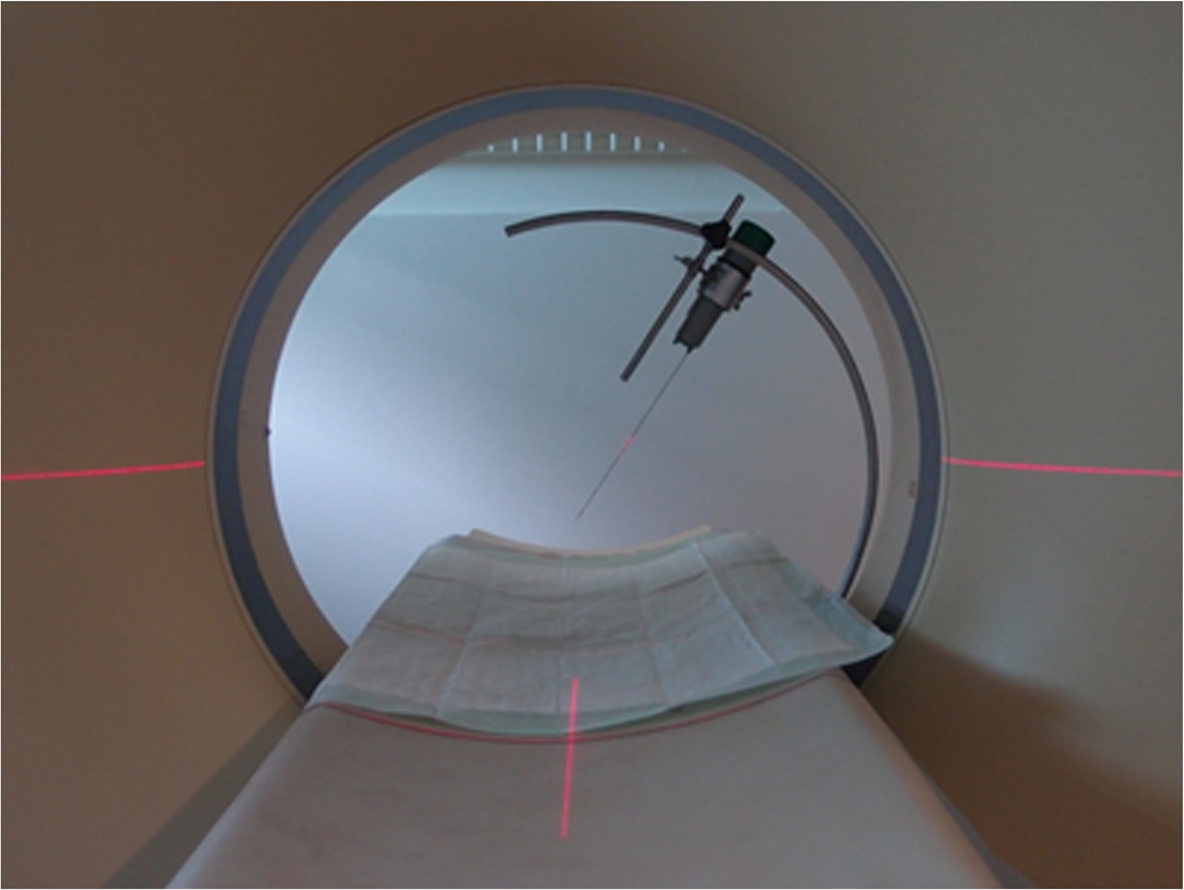
Device for CT-guided core needle biopsy. The device allows free positioning of the biopsy instrument, limited only by the slightly elevated border of the table and the gantry. The biopsy instrument shown is a BARD disposable core biopsy instrument “Monopty”. Other instruments and different kinds of syringes can be attached as well

Biopsy sites were chosen according to suspicious findings, such as enlarged lymph nodes and space-occupying lesions in the lung, liver, spleen or kidney. Disposable core needle biopsy instruments (17 mm sample notch, 16 Ga, 18 Ga, 20 Ga; 18 mm sample notch, 18 Ga and 20 Ga respectively; BARD Disposable core biopsy instruments “Monopty” and “Max Core”) and a manual punch biopsy (14 Ga, thin wall × 15 cm, cannula with centimetre depth markings, 20 mm specimen notch; Pharmaseal Tru-cut biopsy needles) were used. The core needle biopsy instrument was fixed to a self-constructed device attached to the patient table (Fig. 1). The site of entrance through the skin was not shaved, to minimize visibility of the needle track and biasing of the pathologists. With additional scans, the position of the needle tip was controlled and, if necessary, adjusted before taking the biopsy [20–22]. This is called a classic step-and-shot technique [20]. Not every nodule or space-occupying lesion was sampled, because the procedure was rather time-consuming and the availability of the CT room limited. Some organs were sampled despite the lack of a lesion, if affection was suspected. On the other hand, some lesions were detected only after the examination, when the scans were reviewed. If PM-CT examination revealed lesions in bones or in the central nervous system, no CT-guided core needle biopsies were performed. The time span between death and tissue sampling was less than 12 hours in eight and less than 24 hours in two dogs. In dog 10, core needle biopsy was performed 12 days after death.

Between PM-CT and autopsy, cadavers were stored in a refrigeration room at 4–6 °C. The following working day, the cadavers were taken to the Institute of Animal Pathology, together with a written summary of the intra vitam medical history, containing the same information as available for PM-CT. The PM-CT findings were not discussed with the pathologists before autopsy. It was our intention to minimize the influence of the previously performed procedure on the result of autopsy. Taking biopsies inevitably left traces in the skin and the organs, but they were rather discrete due to collapse of the needle tract and the absence of bleeding in the post mortem situation. Only if PM-CT examination revealed lesions in bones or in the central nervous system, the pathologists were informed about their presence beforehand. Autopsy was performed in a routine manner, according to standard protocols, adapted depending on the medical history, clinical symptoms and specific differential diagnoses. The pathologists decided, which organs were to be examined by histopathology. Time spent for this examination ranged between 30 minutes and an hour. In seven dogs, autopsy was performed within the first 24 hours after death. In three dogs, it was performed within four days of death and 15 days after death in dog 10.

Samples of affected organs taken at autopsy and all core needle biopsies were fixed in 10 % buffered formalin and routinely processed for paraffin embedding and histopathology.

Histology of autopsy cases was performed by the pathologist on duty, while all core needle biopsies were investigated by one pathologist (DJW). This pathologist was provided with the same intra vitam medical history as the pathologists responsible for the autopsies plus the information about the biopsy site. She was neither aware of the results of the PM-CT, nor of the autopsy findings. Immunohistochemistry (IHC) was performed on organ samples from autopsy of all eleven dogs and on all core needle biopsies that provided enough tissue for this analysis. Slides derived from paraffin blocks from the biopsy and autopsy samples, respectively, were stained with a panel of CD18, CD3, CD20, mast cell tryptase and cytokeratin. In some biopsies it was not possible to get recuts for the entire antibody panel. In these cases the pathologist selected the most appropriate antibodies to exclude a possible differential diagnosis. Immunohistochemistry was performed with an automated stainer and a standardized protocol was used. A positive and a negative control for each staining were provided.

Findings and diagnoses were evaluated at the following points in time: intra vitam, after PM-CT, after histology of core needle biopsy and after autopsy.

A scoring system was used to describe the reliability of a diagnosis [13]. Information about the cause of death without consultation of a veterinarian was graded with score 1. A diagnosis based on a veterinary examination was graded as score 2. A score 3 diagnosis required additional examination techniques, such as radiography, ultrasonography or blood analysis. Grade 4 was reserved for the most specific test existing for a certain diagnosis.

## Results

### Intra vitam and post mortem findings

In table 1 the medical histories of the eleven BMD are presented (Table 1). The most frequently performed intra vitam tests were thoracic radiography (6 cases), followed by blood analysis (5) and orthopaedic radiography (2). In all dogs, neoplastic disease was on the list of differential diagnoses, but in none of the cases the diagnosis was a definite one.

**Table 1:** Symptoms and Diagnostic Workup intra vitam

Dog	Sex	Age (y)	Medical history	Tests	Diagnostic results	HS	RS
1	F	9	Apathy, dyspnoea, collapse	B, R, U	Anaemia, hypoalbuminaemia; cranial intrathoracic mass; pleural effusion.	S	3
2	F	5.5	Apathy, anorexia, icterus	B, R	Anaemia, hypoproteinaemia, hyperbilirubinaemia; multinodular lung pattern, widened mediastinum.	S	3
3	F	8.5	Paraparesis	R	Pulmonary mass, widened mediastinum.	S	3
4	Fn	9	Apathy, anorexia, dyspnoea	R (m)	Growing pulmonary mass, widened mediastinum.	S	3
5	Mn	6	Weakness, anorexia, collapse, fever	B, U, C	Anaemia, thrombocytopenia, leucocytosis with neutrophilia; mass associated with the spleen, disseminated liver nodules, haemoabdomen.	S	3
6	M	7.5	Anorexia, vomiting	B, R	Anaemia, thrombocytopenia; pulmonary masses, tracheobronchial lymphadenomegaly.	S	3
7	M	6	Splenic mass	P, L, H	Splenic neoplasia, suspicion of HS.	S	3
8	F	7	Lameness	R	Aggressive bone lesions in left femur and tibia.	S	3
9	Fn	12	Weakness, lameness, dyspnoea	N	None.	N	2
10	Mn	3.5	Apathy, anorexia	B, R	Anaemia, thrombocytopenia; intrathoracic mass cranially; abdominal mass effect.	S	3
11	Fn	9	Lameness	R	Aggressive bone lesion in the right humerus.	S	3

This table summarizes the details about the eleven Bernese mountain dogs, their medical history, the diagnostic methods used and their results. The last two columns show the diagnostic quality regarding HS and the reliability score of the diagnosis. For explanation, please consult the methods section of the text

*Abbreviations: y* years, *HS* histiocytic sarcoma and the respective quality of diagnosis, *S* suspicion of histiocytic sarcoma, *N* none, *RS* reliability score [13] *F* female, *M* male, *n* neutred, *B* blood analysis, *R* radiography, *m* multiple consecutive radiographs, *U* ultrasonography, *C* cytology of abdominal fluid from peritoneocentesis, *P* abdominal palpation, *L* explorative laparotomy, *H* histology of splenic sample

Detection of space-occupying lesions intra vitam and post mortem is shown in Table 2. All intra vitam findings could be confirmed by the post mortem techniques.

**Table 2:** Detection of space-occupying lesions

	

The table shows the localization of space-occupying lesions detected intra vitam and post mortem by computed tomography (CT) and autopsy. Intra vitam identification of space-occupying lesions is marked by the first letter of the method used for detection. Colours are used for the post mortem methods CT and autopsy. Lesions and Organs chosen for histology are marked as well. The two columns HS (histiocytic sarcoma) and RS (reliability score) take into account the results of histology that are shown in Table 3

Blue colour: space-occupying lesion detected by PM-CT; purple colour: space-occupying lesion detected in autopsy

R = space-occupying lesion detected by radiography intra vitam; U = space-occupying lesion detected by ultrasonography intra vitam

P = space-occupying lesion detected by palpation intra vitam; L = space-occupying lesion identified in explorative laparotomy

B = CT-guided core needle biopsy of a lesion (blue field) or an organ without lesion (white field), H = lesion or organ chosen for histology at autopsy, A = autolysis severe, precluding assessment; * = splenectomised intra vitam. S = Suspicion of HS; D = definite Diagnosis; N = no diagnosis; h = hours post mortem when CT or autopsy were performed

In all eleven dogs, PM-CT revealed space-occupying lesions. Results of PM-CT and autopsy were similar for lung tissue, thoracic lymph nodes, spleen, kidney, bone, spinal cord and adrenal gland. In the liver, however, autopsy detected space-occupying lesions in seven dogs, whereas such lesions were found in only two dogs by PM-CT (Table 2).

Lesions detected in PM-CT that lacked mentioning in the autopsy reports were: small nodules of 3 mm diameter in the lung of dog 7, enlarged tracheobronchial lymph nodes (left: 20 × 22 × 30 mm, median: 19 × 27 × 24 mm, right: 12 × 18 × 23 mm) in dog 8, enlarged medial iliac lymph nodes in dog 8 (left: 42 × 29 × 79 mm, right: 36 × 30 × 64 mm) and 11 (right: 23 × 20 × 32 mm), nodules in the mesentery in dog 11 (2–7 mm in diameter) and enlarged hypogastric and epigastric lymph nodes in dog 8 (examples of size: gastric lymph node 41 × 28 × 52 mm, jejunal lymph node 29 × 25 × 31 mm).

### Core needle biopsy

Histologic Results of core needle biopsies are presented in Table 3 in comparison to the histologic results of the organ samples gained from autopsy (Table 3). In every dog, IHC confirmed the diagnosis of HS in at least one organ sample from autopsy. Computed tomography-guided biopsies were performed of 23 detected space-occupying lesions and 16 organs without detectable lesions. Of the 39 biopsy sites, samples of 4 sites were lost during processing. The degree of autolysis found in histology is added to the diagnostic result per sample site. Complaints about small sample size, complicating the assessment are noted. Time since death when core needle biopsy and autopsy were performed and the internal body temperature measured during PM-CT are included in the table as well.

**Table 3:** Histologic results of biopsy and autopsy regarding identification of histiocytic sarcoma

	

In row B you can find the assessment of the samples taken by CT-guided biopsy, arranged by biopsy site and in row H the assessment of the corresponding organ samples taken in autopsy. For each sample site the diagnostic quality of the biopsies regarding histiocytic sarcoma (HS) is shown in the first of three fields. The second field informs about the degree of autolysis (1 = slight, 2 = moderate, 3 = severe) of the samples. In the third field, complaint about very small sample size is noted, whenever the case. Information with influence on the cadaveric alteration status is given in the third and fourth column. Body temperature was measured with a temperature probe within the liver after the performance of the first CT scan and left in place during CT-guided biopsy. The last column contains the reliability score of the diagnosis of HS (2 = based on an examination by a veterinarian; 3 = including additional diagnostic methods; 4 = based on the most specific test available)

Orange colour: suspicion of histiocytic sarcoma, no immunohistochemistry possible because of lack of sufficient tissue; brown colour: diagnosis of histiocytic sarcoma based on histology; green colour: diagnosis of histiocytic sarcoma based on histology and immunohistochemistry. N = No identification of neoplastic cells possible; C = complaint about small size of tissue sample; L = biopsy sample lost during processing

Histologic assessment of 35 organs yielded a definitive diagnosis of HS based on histology and IHC for six biopsy sites (lung: 4, spleen: 1, liver: 1). A definitive diagnosis of HS could be made based on histology of core needle biopsy in five other sites (lung: 2, thoracic lymph node: 2, spleen: 1) but due to insufficient sample size, IHC could not be performed. For six sites, the final diagnosis was a suspicion of HS. Identification of neoplastic cells was not possible in the samples of the remaining 18 sites (lung: 2, thoracic lymph node: 1, spleen: 3, liver: 7, kidney: 5). Among these, samples of eleven sites were severely autolytic. The remaining seven sites with moderate autolysis included four sites with only small samples.

In summary, PM-CT combined with CT-guided biopsies yielded a definitive diagnosis of HS in ten of eleven dogs. In the remaining dog 1, no neoplastic cells could be assessed. Regarding the reliability score, the procedure achieved the maximal score of 4 in six of eleven dogs.

## Discussion

The intra vitam findings in these eleven BMD, especially enlarged lymph nodes, pulmonary masses and anaemia are consistent with the clinical findings reported in other publications about disseminated HS [11, 16, 19, 23]. None of the dogs had a definitive diagnosis intra vitam. In our experience, the reason for this is, that in the routine clinical work up, the combination of characteristic clinical signs, the poor general condition and the breed affiliation lead to the strong assumption of an affection by HS. Therefore, dogs are often euthanized without a definitive diagnosis based on the morphological assessment of lesions. However, if the cause of death of a BMD shall be considered for the purpose of breeding selection, for the evaluation of genetic tests and breeding value estimations, a definitive diagnosis is essential.

Post mortem computed tomography turned out to provide good results regarding detection of space - occupying lesions in the lung, lymph nodes, spleen, kidneys and bone. It revealed space-occupying lesions in all eleven dogs. This high sensitivity is similar to native CT studies in living animals and is based on good contrast to the unaffected surrounding tissue (air filled lung parenchyma, fat), the destruction of characteristic organ architecture (kidney) and the excellent resolution in bone tissue [24]. The radio-density dependent visualization of the body in CT is advantageous for the detection of lymph nodes within the fat [24]. In the slender shaped spleen, space-occupying lesions protrude over the regular surface and are thus readily detectable [24].

In the liver however, the lack of peritoneal fat between the lobes limits the assessment of the lobar surfaces due to border effacement [24]. This might explain the fact that space-occupying lesions were missed by PM-CT. In three cases, hepatic nodules described in the autopsy report were of considerable size and one would have expected them to be easily detectable by CT. Hence, with the scanning protocol used in this study, absence of space-occupying lesions in the liver does not exclude the presence of neoplasia. In the living animal, assessment of the liver parenchyma in CT is complemented by administration of contrast media [24]. However, lack of blood circulation after death complicates this procedure in many ways. The Virtopsy project in human medicine has developed a method of PM whole body angiography [25]. With the aid of a modified heart lung machine, a circulation is established and a dynamic angiography can be performed, showing the arterial, parenchymatous and venous systems [25]. In order to test a minimally invasive economic screening program for post mortem diagnosis we decided for a native general purpose PM-CT examination. In retrospect, an economically viable solution would have been the adaptation of the scanning protocol: reduction of photon energy and increase of photon quantity improves soft tissue contrast in radiography and therefore also in CT. Such an optimization might have resulted in higher sensitivity for liver nodule detection.

**Fig. 2:**
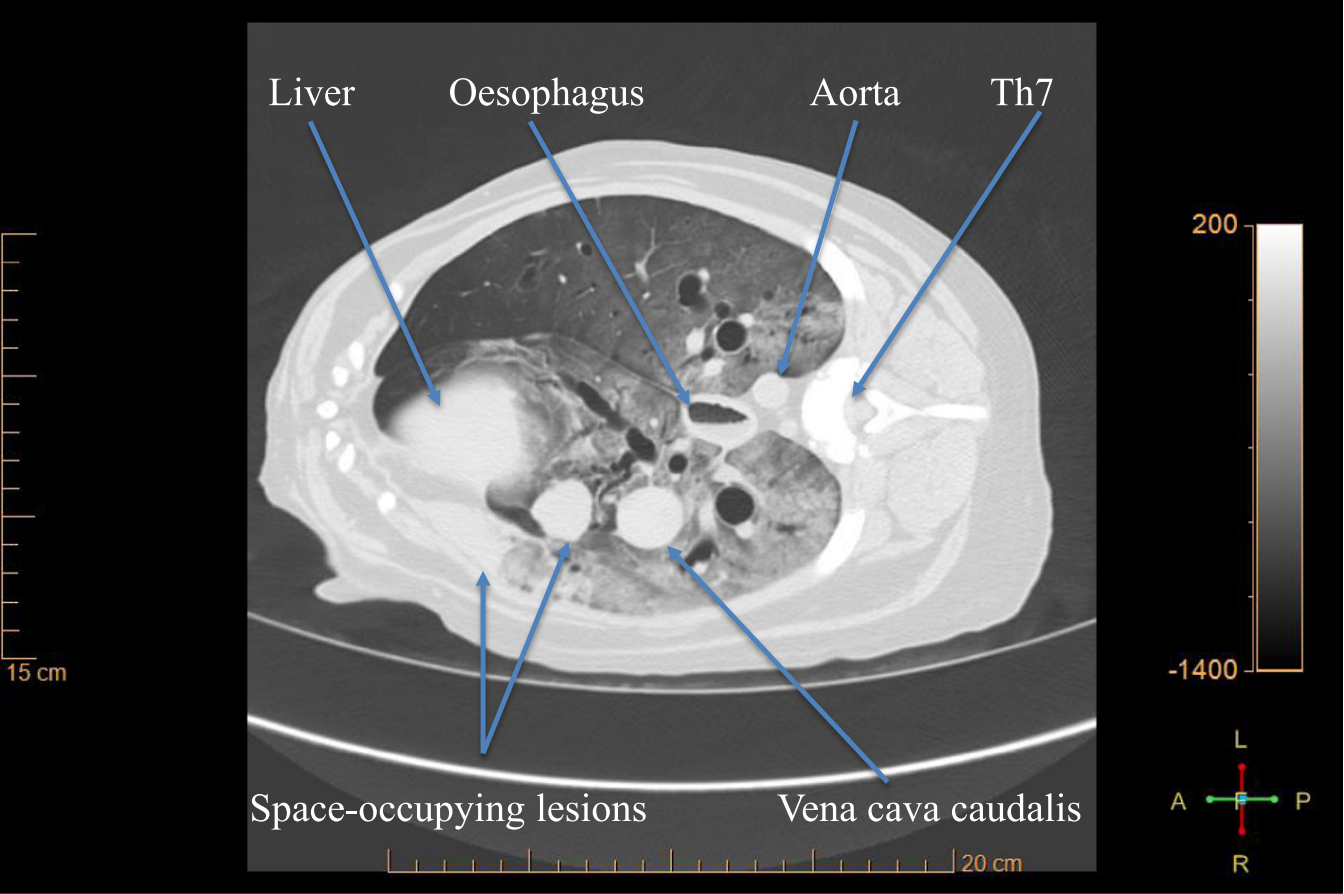
Post mortem lung with space-occupying lesions. Transverse CT-slice of the thorax of dog 9, at the level of the 7^th^ thoracic vertebra. The gravity-dependent gradual increase in ground-glass opacity in the lung tissue reflects the passive distribution of air and blood after death. It is typical for the post mortem lung [26]. Space-occupying lesions are visible in the accessory and in the right middle lung lobe. For the latter lesion, atelectasis is an important differential to neoplasia

In the lung, detection of lesions was more challenging in areas of ground-glass opacity or consolidation, which is a combination of PM collapse and oedema [26]. Pulmonary vessels as well as space-occupying lesions were discernible, if the surrounding pulmonary tissue contained enough air (Fig. 2). In some cases, entire lung lobes were infiltrated by soft tissue dense material replacing the air containing lung parenchyma. The pulmonary findings in dog 1 demonstrate the limitations of lesion demarcation using just a native PM-CT protocol. Comparison with the results of autopsy revealed, that different pathological areas of similar radio-densities, namely atelectatic lung, pulmonary mass lesions and haemorrhagic pleural effusion with blood clots were in contact. They cannot be differentiated with certainty from each other because of border effacement. The assessment of the lung could be improved in such cases either by repeating the scan with an optimized examination protocol, as described above, or by using ventilation technique [26].

In autopsy, lesion detection depends on the localisation (close to the surface vs deep in the parenchyma), colour, consistency and size. Not all lesions detected in PM-CT were detected in autopsy. This can be explained by the fact, that we have chosen a routine autopsy, performed within 30 to 60 minutes. It was not the aim to detect every single nodule. Some were localised deeply within the parenchyma. There, the cross sectional way of assessment in CT is advantageous for small nodular lesions. Enlarged thoracic and medial iliac lymph nodes were overlooked in autopsy in two cases each. Detection of enlarged lymph nodes within a large amount of fat can be difficult in autopsy, whereas the different densities of fat and lymph node are obvious in CT.

Certain properties of the two methods compared are complementary. Autopsy can profit from CT information in cases of bone lesions, lesions in the spinal canal, intracranial lesions and presence of foreign material [1]. Knowing the localization of a lesion before starting the dissection reduces the probability to destroy it by chance [1] or to miss it .

An important advantage of autopsy is the possibility to select the tissue for histopathology by visual assessment. For internal organs this process is by far less time-consuming than our classic step-and-shot technique of CT-guided tissue sampling [20]. Robotic means for tissue sampling, as developed for the Virtopsy project [27] would be precise and fast. The dissection in autopsy is also of advantage for sampling of special sites, as for example lesions in the bone morrow or of the bone itself. For economic reasons we did not sample such lesions during PM-CT. But since they might have been overlooked in autopsy, radiologists and pathologists had agreed before starting the study to communicate this information before autopsy.

The target diagnostic reliability score of 4 was achieved in six of eleven dogs by PM-CT combined with core needle biopsy. A reliability score of 3 was achieved in the following cases: in four dogs, histology of core needle biopsy came to a definite diagnose of HS, but immunohistochemistry could not be performed due to lack of sufficient tissue; in the remaining dog, the high degree of autolysis made a definitive diagnosis impossible (Table 3).

In our study, reasons for ambiguity of histological diagnosis of core needle biopsies were a high degree of autolysis and/or a very small sample size, precluding IHC in several samples. Autolysis is attributable to the process of putrefaction that develops rapidly in the liver. Central tumour necrosis is another relevant process that may be present independent of death and leads to the same difficulties in histology. In some biopsy samples taken from the centre of larger masses, the high degree of autolysis was obvious, because they were dissolving on the needle. Such samples were discarded and sampling was repeated. In other cases however, autolysis was only recognized in histology. In order to avoid the necrotic centre, we preferred to sample the outskirts of the space-occupying lesions. This strategy turned out to be disadvantageous in small pulmonary lesions, however. In dog 2 for example, PM-CT showed pulmonary nodules of up to 12 mm in diameter. However, autopsy revealed pulmonary nodules of 2 to 6 mm in size and in histology, neoplastic cells were surrounded by atelectatic pulmonary tissue. A possible reason for the biopsy result of slight interstitial pneumonia without presence of neoplastic cells in this case could be, that an atelectatic part surrounding the nodule had been sampled. This difference between consolidated lung tissue and neoplastic nodule is not evident in CT [24]. PM angiographic and ventilation technique might be useful to differentiate between intact and necrotic tissue.

Since histology is the only way to achieve a definite diagnosis of HS, improvement of tissue sampling is essential for the future. Multiple, large sized core needle biopsies from different parts of a space-occupying lesion detected in CT are required. A needle of at least 14 Ga should be used, as recommended for PM core needle biopsy by Aghayev [5] and for lesions near the body surface, an excisional biopsy should be considered. Furthermore, core needle biopsies should be frozen to allow optimal processing for IHC and diagnosis of HS.

Efforts should be made to keep time from death to examination and sampling as short as possible, with refrigeration during storage and transportation, if possible. We deliberately included dog 10 in this study to show, that a diagnosis was possible despite the long term post mortem.

## Conclusions

Based on the examinations of these eleven BMD with HS, we conclude that PM-CT is a useful modality for the detection of space-occupying lesions. The study shows, that native PM-CT in combination with CT-guided core needle biopsy can provide a definitive diagnosis of HS in BMD. Routine autopsy was superior to this minimally invasive technique, though. Improvement of the biopsy technique and the scanning protocol will increase the probability of a definitive diagnosis of HS.

## Abbreviations

BMD: Bernese mountain dog
HS: Histiocytic sarcoma
IHC: Immunohistochemistry
PM-CT: Post mortal computed tomography
